# Profil épidémiologique et clinique des consommateurs de cannabis au centre de soins d’accompagnement et de prévention en addictologie de l’Hôpital Laquintinie de Douala, Cameroun

**DOI:** 10.11604/pamj.2023.44.143.38666

**Published:** 2023-03-22

**Authors:** Hugo Hermann Divahe Bohongwe, Christian Eyoum, Jonas Guy Atchou Basseguin, Anne Mbong Andong, Lydie Foko Mamguem, Pechel Dongmo Tsague, Erero Njiengwe, Laurent Karila

**Affiliations:** 1 Centre de Soins, d’Accompagnement et de Prévention en Addictologie, Hôpital Laquintinie de Douala, Douala, Cameroun,; 2 Faculté de Médecine et des Sciences Pharmaceutiques, Université de Douala, Douala, Cameroun,; 3 Centre d´Enseignement, de Recherche et de Traitement des Addictions, Hôpital Universitaire Paul Brousse, Université Paris Saclay, UR PSYCOMADD, Paris, France

**Keywords:** Cannabis, profil clinique, Douala, Cannabis, clinical profile, Douala

## Abstract

Le cannabis est la drogue illicite la plus consommée au monde. Elle est consommée par les tranches d’âge dont les principales sont les adolescents et les jeunes adultes. Sa consommation engendre des complications somatiques, psychiatriques et sociales. Les données dans notre contexte sont rares. Le but de notre travail était de décrire le profil épidémiologique et clinique des patients présentant une addiction au cannabis au Centre de Soins, d’Accompagnement et de Prévention en Addictologie de l’Hôpital Laquintinie de Douala. L’analyse rétrospective portait sur les patients suivis de mars 2021 à juillet 2022 pour addiction au cannabis. Sur les 45 cas d’addiction au cannabis, 44 (98%) patients de sexe masculin et l’âge moyen était 21,97 ans, la tranche d’âge la plus représentée est comprise entre 20 et 24 ans (28/44 soit 63%); quarante neuf (49%) des consommateurs sont des étudiants, 62% des mères accompagnant le patient en consultation. L’âge de début de consommation est 16 ans (31%), la forme de cannabis la plus consommée est l’herbe (100%) et 100% des patients consomment par voie inhalée (fumée). La complication la plus fréquente était le syndrome amotivationnel (31%). L’âge de début de consommation est précoce. L’herbe est la forme la plus consommée et se fait par voie inhalée (fumée). Les complications les plus fréquentes sont le syndrome amotivationnel, les troubles cognitifs les troubles du sommeil et le syndrome de sevrage.

## Abstract (en)

Cannabis is the most widely used illicit drug in the world. It is consumed by age groups, the main ones being adolescents and young adults. Its consumption leads to somatic, psychiatric and social complications. Data in our context are scarce. The purpose of our work was to describe the epidemiological and clinical profile of patients with cannabis addiction at the Centre for Care, Support and Prevention of the Laquintinie Hospital in Douala. We conducted a retrospective cross-sectional study of patients followed from March 2021 to July 2022 for cannabis addiction at the Addiction Care, Support and Prevention Center of the Laquintinie Hospital in Douala. The diagnosis of use disorder was based on a dependency syndrome related to cannabis use (single event usage). Data entry and analysis were performed using SPSS version 7.1 software. Of the 45 cases of cannabis addiction, 44 (98%) were male patients with an average age of 21,97 years. The most affected age group was between 20 and 24 years (28/44 or 63%); 49% of consumers were students, 62% of mothers accompanying patient to the consultation. The age of cannabis use initiation was 16 years (31%), the most commonly used form of cannabis was herbal (100%) and 100% of patients used inhalation (smoking). The most common complication was amotivational syndrome (31%). Cannabis use initiation occurs at an early age. The most commonly used form of cannabis is herbal administered through inhalation (smoking). The most common complications are amotivational syndrome, cognitive disorders, sleep disorders and withdrawal syndrome.

## Introduction

L´addiction est un trouble caractérisé par une consommation répétée de substance psychoactive licite (tabac, l´alcool, les médicaments psychotropes) ou illicite (cannabis, cocaïne, amphétamines comme le 3,4-méthylènedioxy-N-méthylamphétamine (MDMA) ou la méthamphétamine, nouveaux produits de synthèse …) ou à un comportement (sexe, jeux de hasard et d´argent, réseaux sociaux, jeux vidéo …) est définie comme une trouble caractérisé par un processus récurent, comprenant un phénomène de consommation répétée d´intensité variable puis l´installation progressive d´une dépendance physiologique s´accompagnant de signes de tolérance et/ou de sevrage, d´un craving (envie irrépressible de consommer), d´une perte de contrôler, d´un déni et de la recherche de la substance malgré les risques médicaux, psychologiques, psychiatriques et sociaux encourus et connus. Le caractère chronique et les rechutes sont caractéristiques de ce trouble. Cette pathologie peut concerner les comportements également [1]. De la consommation de substances psychoactives illicites on note que le cannabis est la drogue illicite la plus consommée au monde. Globalement, son utilisation semble augmenter, avec une estimation de 162 millions (4%) des adultes du monde qui l´utiliseraient en 2004 [2]. Pour lutter contre l´usage de cette substance illicite, il existe des structures de consultation hospitalières et extrahospitalières d´addictologie (centres de soins, d´accompagnement et de prévention en addictologie-CSAPA) en 2008 en France. Dans ces centres on a noté entre 2007 et 2014 une prévalence de la consommation de cannabis de 18%, la proportion des patients pris en charge pour consommation de cannabis dans les CSAPA est évaluée à 40-50% [3]. Au Cameroun, une étude menée au sein de l´hôpital Jamot à Yaoundé sur le profil sociodémographiques et les comorbidités des usagers en consultation d´addictologie à Yaoundé a révélé une prévalence de 72,4% pour le cannabis [4]. En vue de connaitre la particularité du trouble lié à l´usage de la consommation de cannabis dans le contexte de prise en charge en milieu hospitalier, nous nous proposons d´étudier le profil épidémiologique et clinique des consommateurs de cannabis au Centre de Soins d´Accompagnement et de Prévention en Addictologie (CSAPA) de l´Hôpital Laquintinie de Douala.

## Méthodes

**Type et conception de l´étude**: il s´agissait d´une étude transversale retrospective. L´étude s´est déroulée du 01^er^ février 2022 au 30 septembre 2022 soit 08 mois, avec une période de recrutement allant de mars 2021 à juillet 2022. Il s´agissait d´une étude effectuée au sein du Centre de Soins, d´Accompagnement et de Prévention en Addictologie (CSAPA) de l´Hôpital Laquintinie de Douala (HLD). Etait inclus tout dossier médical de patient suivi au CSAPA de l´Hôpital Laquintinie de Douala avec un trouble de l´abus de substance lié au cannabis dont le diagnostic avait été posé entre durant la période allant de mars 2021 à juillet 2022.

**Critères d´inclusion**: tout dossier médical de patient suivi au CSAPA de l´Hôpital Laquintinie de Douala avec un trouble de l´abus de substance lié au cannabis dont le diagnostic avait été posé durant la période allant de mars 2021 à juillet 2022.

**Critères d´exclusion**: les dossiers médicaux incomplets ont été exclus de l´étude.

**Échantillonnage**: la technique d´échantillonnage utilisée a été non probabiliste basée sur le recrutement exhaustif des dossiers obéissant aux critères d´éligibilité.

**Collecte de données**: les données ont été consignées dans un questionnaire prévu à cet effet avec recueil des données recherchées à l´admission et au suivi étaient: a) **les comorbidités**: somatiques, psychiatriques et addictologiques; b) **cliniques**: durée d´évolution des symptômes du trouble avant la première consultation, la forme de cannabis consommée, la voie d´administration, les signes physique et psychique prédominants, de début des premières consommations, forme clinique de la maladie, les complications; c) **le suivi thérapeutique**: traitement initial reçu, régularité du suivi et amélioration clinique.

**Analyse des données**: la saisie et analyse des données ont été faites grâce au logiciel SPSS version 7.1. Les statistiques descriptives ont été appliquées pour condenser et ordonner les variables. Les variables quantitatives ont été exprimées par la médiane avec les extrêmes et/ou la moyenne accompagnée des écart-type. Les variables qualitatives ont été représentées sous formes de graphiques. Les tests de Khi-2 et de Fisher ont été utilisés pour évaluer l´association entre deux variables qualitatives. Le seuil de signification statistique a été fixé à 0,05.

## Résultats

**Caractéristiques sociodémographiques**: notre population d´étude était constituée d´un (02%) patient de sexe féminin et 44 (98%) patients de sexe masculin. La tranche d´âge la plus représentée était celle comprise entre 20 et 24 ans (63,6%). L´âge moyen des consommateurs de cannabis dans notre échantillon était 21, 97 ans. Quarante-neuf pourcent (49%) des patients étaient des étudiants. Soixante-deux pourcent (62%) de la demande des soins est faite par les mères accompagnant leur patient.

**Caractéristiques cliniques**: trente-et-un pourcent (31%) des patients ont débuté la consommation à l´âge de 16 ans, 100% des patients consomment du cannabis sous forme d´herbe, 100% (45) des patients consomment du cannabis par voie inhalée (fumée), 4% (02) des patients consomment du cannabis par voie enterale et 4% (02) des patients par voie transcutanée (huile) 31% des patients ont manifesté comme complication de la consommation de cannabis un syndrome amotivationnel.

## Discussion

L´âge moyen de nos patients était 21,97 ans. Nos résultats se rapprochent de ceux de l´Observatoire Français des Drogues et des Toxicomanes (OFDT) qui a rapporté un âge moyen pour les consommateurs de cannabis à 27,1 ans [3] et ceux d´Guillem E. *et al*. [5]. La tranche d´âge la plus représentée dans notre échantillon était celle des 20 à 24 ans (63,6%). Résultat similaire à ceux de Mbongo´o *et al*. [4]. Mais différents de celui de Salifou *et al*. qui a trouvé une tranche d´âge comprise entre 25 à 34 ans [6]. Plusieurs auteurs ont rapporté une prédominance du sexe masculin dans leurs études [4,5]. Notre série comportait 98% de sexe masculin. Bien que cette prédilection masculine semble une constance, la raison précise est inconnue. De plus en plus, les femmes présentent un trouble de l´usage de substance [3,5]. Dans notre étude, il ressortait que le trouble de l´usage lié à la consommation de cannabis touchait plus les jeunes scolarisé précisément les étudiants (48,9%). Certains auteurs ont effectué des études communautaires [6-8]. Nikiéma *et al*. [7] ont effectué leur étude en milieu scolaire de même que l´OFDT [8] où la consommation de cannabis est très forte. D´autres secteurs d´activités ont été rapporté comme le secteur automobile, et aussi dans les industries chimiques, pétrochimique, métallurgique, les métiers du bâtiment, dans le secteur du transport et dans le secteur médical et militaire [6,9]. Du reste il faut noter que la proportion des consommateurs après les élèves (24,14%) était sans profession (17,8%).

Soixante-deux pourcent (62%) des consommateurs se sont présentés en consultation accompagné de leur mère, résultat similaire à ceux de Mbongo´o *et al*. [4], mais différent de ceux d´Guillem E. *et al*. chez qui le patient seul était majoritairement demandeur de soins suivi de ceux accompagnés des parents [5]. Cela pourrait s´expliquer par le contexte socioéconomique où la majorité des personnes sont rapidement autonome et très vite engagé dans la vie socioprofessionnelle. Ce qui est très différent de notre contexte où la majorité des consommateurs sont issus de famille monoparentale dans un contexte où les jeunes femmes sont mères célibataires précocement et où il manque une figure paternelle. Notre échantillon a rapporté un âge de début de consommation observé de façon rétrospective à 16 ans suivi de ceux ayant commencé à l´âge de 17 ans. Beck *et al*. dans une étude rétrospective sur la sociologie et épidémiologie des consommations de substances psychoactives a retrouvé en fonction des substances consommées un âge moyen de début de consommation de 14 ans et donc 17 ans pour les consommateurs de cannabis [10], résultats similaires à ceux que nous avons retrouvés. Par contre l´ODFT a rapporté un âge de début d´expérimentation de la consommation de cannabis de 15 ans et un pic à 17 ans [8]. Cet âge précoce de consommation de substances psychoactives et en particulier le cannabis peut s´expliquer par le fait que cette période constitue l´adolescence, une période marqué par des changements sur le plan physique, psychique et hormonal, se caractérisant par un besoin de s´affirmer vis-à-vis de ses pairs, de se construire une identité différente des adultes, où les messages interdicteurs ou préventifs sont mis en doute, un sentiment d´immortalité avec besoin de l´éprouvé et des sensations fortes et du risque pour exister complété par le besoin d´apprivoiser un corps mouvant. Les formes de cannabis consommées le sont en fonction de la teneur en tétra-hydrocannabinnol (THC) qui est plus concentrée dans l´huile de cannabis (30-80%) de THC, suivi de la résine (26,5%) et l´herbe (5-22%) selon l´OFDT [8]. Dans notre échantillon, tous les usagers étaient consommateurs de cannabis sous forme d´herbe, résultat proche de ceux de l´OFDT en 2014 [3]. Cela peut s´expliquer dans notre contexte par le fait que le cannabis pousse facilement dans nos climats, facile à sa culture.

Plusieurs auteurs ont rapporté un mode de consommation par voie inhalée [6-8], dans notre échantillon tous les usagers consomment du cannabis par voie inhalée (fumée), résultat similaire rapporté par Guillem E. *et al*. [5] et qui peut être confirmé par une étude menée Benard *et al*. sur la conduite à tenir face à une consommation de cannabis en médecine générale [11]. Les complications résultant d´une consommation chronique de cannabis sont le syndrome de sevrage au cannabis, les troubles cognitifs, le trouble anxieux aigu, un épisode dépressif caractérisé, un syndrome amotivationnel entre autres [11]. Nous avons rapporté comme complication majeure dans notre échantillon le syndrome amotivationnel (31%), résultat différents de ceux rapportés par Guillem E. *et al*. qui rapporte une prédominance des troubles de l´humeur suivi des troubles anxieux et des troubles du comportement alimentaire [5] cela peut s´expliquer par le fait que dans son échantillon parmi les usagers de cannabis plus des deux tiers (73%) ont un antécédent de suivi psychologique ou psychiatrique avec ou sans rapport avec l´usage de substances psychoactives.

## Conclusion

Au terme de notre étude, il ressort que l´âge de début de consommation est de 16 ans, les patients atteints de trouble de l´usage de substance lié au cannabis sont des jeunes pour la majorité scolarisés, la forme de cannabis la plus consommée est l´herbe et elle se consomme par voie inhalée (fumée). Les complications les plus fréquentes liées à la consommation de cannabis sont le syndrome amotivationnel, les troubles cognitifs, les troubles du sommeil et le syndrome de sevrage.

### Etat des connaissances sur le sujet

- *Le cannabis est la drogue illicite la plus consommée au monde;*
- *Sa consommation engendre des complications somatiques, psychiatriques et sociales*.

### Contribution de notre étude à la connaissance

- *Permet de faire la description clinique des consommateurs de cannabis vus dans les centres de soins, d´accompagnement et de prévention en addictologie;*
- *Permet de faire une mise au point du profil épidémiologique des consommateurs de cannabis vus dans les centres de soins, d´accompagnement et de prévention en addictologie;*
- *Répertorier les complications les plus fréquentes pour envisager une meilleure prise en charge*.
